# Reactive, Inelastic, and Dissociation Processes in Collisions of Atomic Nitrogen with Molecular Oxygen

**DOI:** 10.1021/acs.jpca.0c09999

**Published:** 2021-04-28

**Authors:** Fabrizio Esposito, Iole Armenise

**Affiliations:** CNR ISTP (Istituto per la Scienza e Tecnologia dei Plasmi), Via Amendola 122/D, 70126 Bari, Italy

## Abstract

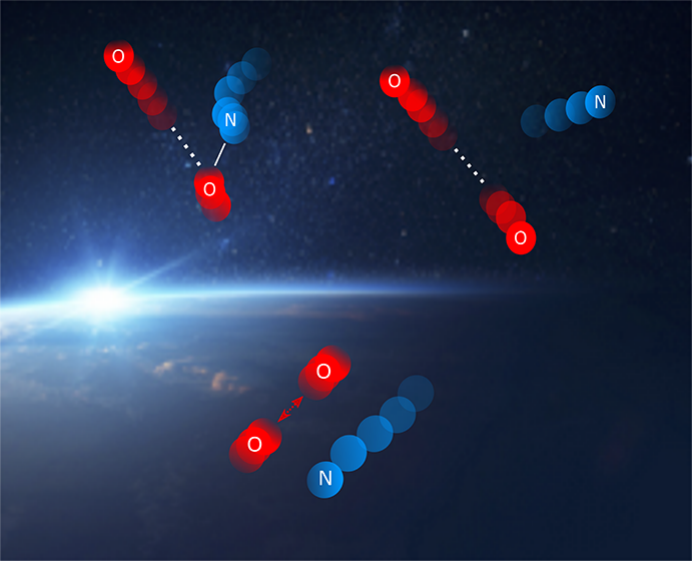

Collisions of atomic nitrogen with molecular oxygen have been treated with the quasiclassical trajectory method (QCT) in order to obtain a complete database of vibrationally detailed cross sections and rate coefficients for reactive, inelastic, and dissociation processes. For reaction rate coefficients, the agreement with experimental and theoretical data in the literature is excellent on the whole available interval 300–5000 K, with reliable extension to 20,000 K. For the inelastic case and for dissociation, no comparisons are available; therefore, a study of QCT reliability is proposed. In the inelastic case, it is found that “purely inelastic” and “quasireactive” collisions show not only different mechanisms but also different QCT levels of reliability at low energy. For dissociation, similar considerations bring to the conclusion that for the present collisional system, the QCT method is appropriate on the whole energy range studied. Rate coefficients for all the processes studied are provided in the electronic form.

## 1 Introduction

Air species in vibrational nonequilibrium are currently considered in many theoretical and experimental studies regarding different fields of great technological interest, such as combustion,^1^ air plasmas,^2−4^ space weather,^5,6^ shock waves,^7−10^ hypersonic flight,^11−13^ and plasma medicine.^14−17^ In these problems, successfully investigated by means of a state-to-state kinetic approach, the collisions of atomic nitrogen with molecular oxygen are of large interest. In the combustion community, for example, the Zeldovich mechanism is well known:
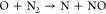
1
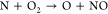
2for the formation of nitrogen oxide from oxygen and nitrogen combinations of ground-state atoms and diatoms. However, when vibrational nonequilibrium^18,19^ is taken into account, eqs 1 and 2 become the much more challenging set
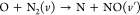
3
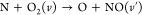
4with *v* and *v*′ being the initial and final vibrational quantum numbers, respectively. As a consequence, even the detailed internal energy exchange processes must be considered because they alter the vibrational distribution
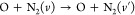
5
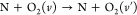
6

If sufficient total energy is available, even state-selected dissociation comes into play
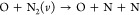
7
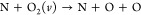
8

In a previous paper,^20^ we have calculated the processes 3-5-7 concerning O + N_2_ collisions and made them computationally available for kinetic models by means of suited interpolations. Now, we complete the picture by presenting our results about the processes 4-6-8 concerning N + O_2_ collisions, in a similar fashion as in ref (20), making available in the Supporting Information the rate coefficients as a function of initial and final vibrational states and temperature. Calculation of complete databases of dynamical quantities to be used in models is a big challenge, for the large ranges of total energy required, which implies the careful evaluation of the suited dynamical methods (even more than one) to be used. The quasiclassical trajectory method (QCT) is nowadays popular for solving this kind of problems, considering its good general reliability and the possibility of easily distributing computations into parallel and/or gridded computer networks. However, one should be well aware of the limits of application of the dynamical method used, in order to perform calculations always in the best conditions for both the problem and the method. In refs,^20,24^ these issues concerning QCT are studied in some representative cases, with some comparisons with highly accurate calculations obtained by computationally expensive quantum mechanical (QM) methods. The conclusions of those studies are also applied here for justifying the dynamical method chosen and in some cases for delimiting its range of application. Particular attention has been devoted to calculation of reaction rates in the range 300–1000 K because of their importance in upper atmosphere kinetics.^6^ It is shown that the present approach is able to gain results in remarkable agreement with experimental data available.

## 2 Method of Calculation

The calculations in this work have been performed with the standard quasiclassical method with histogram binning, using a variable time step driven by trajectory error analysis.^25^ The well-known and accurate potential energy surfaces (PESs) ^2^A′ and ^4^A′ of ref (26) correlating with ground-state reactants and reactive products have been used. The reaction path from N + O_2_ to O + NO is exothermic by about 1.4 eV, but there is a small barrier to the reaction of about 0.3 eV for ^2^A′ PES and about 0.6 eV for ^4^A′. The initial states considered include all the vibrational states (*v* = 0–43) supported by the O_2_ diatomic potential present in the PESs used here, with one in 15 rotational state *j* from the set associated with each vibrational state *v* (one in 5 for *v* ≥ 35 and all *j* for *v* ≥ 42), plus the highest *j* value for each *v*. A linear interpolation of cross sections on *j* values is then used to reconstruct the whole set, with quite good accuracy on integrated rate coefficients starting from a temperature *T* of at least 1000 K, as proved by some specific full-*j* calculations in refs (20) and (21) and in the present work. The number of trajectories calculated on each PES is more than 6 × 10^5^ per initial rovibrational state. For correctly treating the low-temperature reactive results of interest in thermosphere studies (see below), an additional set of trajectories calculated only on ^2^A′ PES (the only useful at low *T* for reaction) has been added from specific initial states: *v* = 0–2 with *j* = 1–29 (including only odd *j* numbers, as usual for O_2_ molecules), using 110 million trajectories. All the cross sections are calculated in the collision energy range: 0.001–10 eV with a continuous, uniform distribution, in order to obtain an accurate determination of thresholds of the various processes considered in this work. Cross sections instead of rate coefficients calculations are important for their use into models that include translational nonequilibrium, as in the case of direct simulation Monte Carlo codes. Suited integration of the present calculated cross sections yields the state-to-state rate coefficients, to be used in master equation studies in which only rovibrational nonequilibrium is studied. A direct calculation of rate coefficients instead of cross sections would be computationally cheaper, but recovering cross sections from rate coefficients is a difficult task,^27^ solvable only within some model approximations.

## 3 Results

### 3.1 Reaction

**Figure 1:**
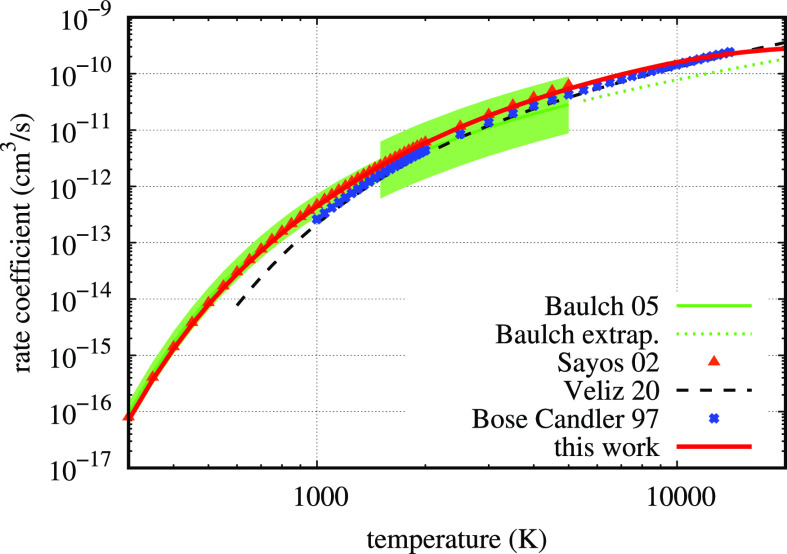
Comparison of the thermal rate coefficient of process (2) from the present work as a function of temperature with the extensive literature review of experimental results in ref (28) (Baulch 05). The green shaded area is the error estimate on experimental data, while the dotted line is the fit extrapolation. The other theoretical results are from refs (26) (Sayós 02),^34^ (San Vicente Veliz 20), and^33^ (Bose Candler 97).

**Figure 2:**
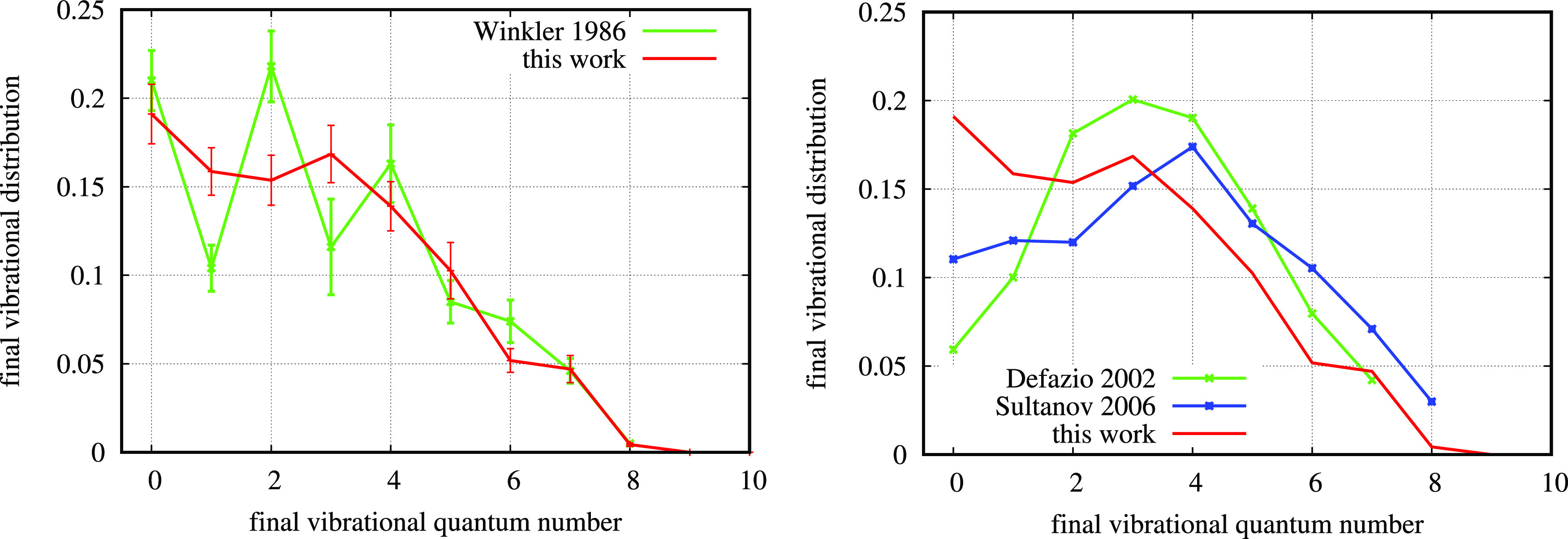
Left panel: Comparison of final vibrational distributions of the reaction of N + O_2_ → NO(*v*′) + O at *T* = 300 K as obtained using the QCT method (this work) with error bars and from experimental values from ref (29); Right panel: comparison of present work with data from ref (39) (wave packet) and from ref (38) (time-independent quantum method).

**Figure 3:**
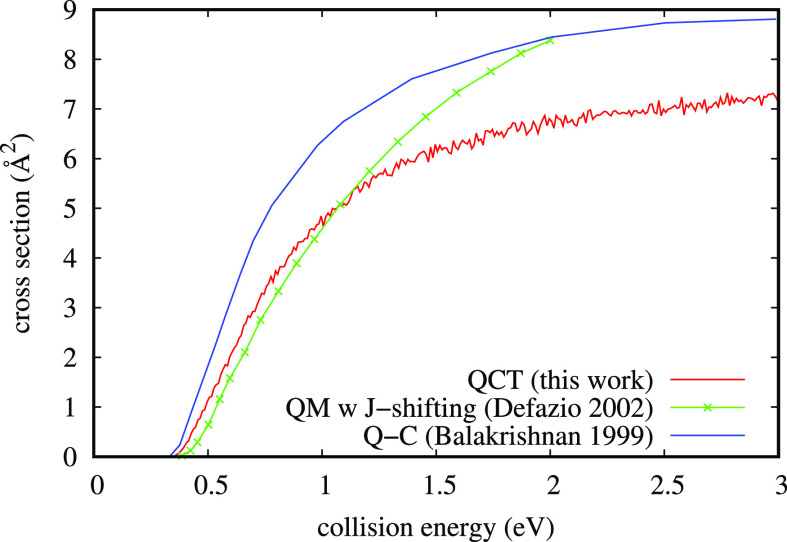
Comparison of the reactive cross section from O_2_ (*v* = 0, *j* = 1) as obtained on the same PES with QCT in this work and by ref (39) (Defazio 2002) with the approximate wave packet method and on a different PES by ref (42) with a quantum-classical method (Balakrishnan 1999). In this last case, the reaction barrier is lower.

The thermal reactive rate coefficient has been calculated in this work using both ^2^A′ and ^4^A′ PESs with the suited degeneracy factors (1/6 and 1/3, respectively, see also the section on dissociation). Thermal rate coefficients are obtained in this work as a Boltzmann average over state-selected rate coefficients, in turn calculated by integration of cross sections. In Figure 1, there is a comparison of the calculated rate coefficient (red curve, with statistical errors of the same size of curve width) with the best fit from Baulch et al.^28^ (green curve) of a collection of twelve experimental data sets covering the temperature interval 280–5000 K, including an estimate of its reliability (the green shaded area). These data from Baulch et al.^28^ represent a recommended value for the reaction rate of the present system and are used in many models. The comparison is excellent on the whole common interval. At room temperature, however, the fit is more uncertain, due to the scatter in experimental data. To better investigate this point, Table 1 presents some relevant experimental results at room temperature in comparison with the present QCT result and the variational transition state with tunneling correction (ICVT/μOMT) of ref (26). The QCT result is lower by an amount from 1.5% with the Winkler et al. data^29^ to 24% with Barnett et al. data^30^ with respect to experimental values (excluding the result by Clark and Wayne,^31^ clearly too large in comparison with all the most recent data from experiments) and by 8% with ICVT/μOMT of ref (26). It is worth noting that for reaching this level of accuracy, the additional set of trajectories has been important and that limiting initial rovibrational states to *v* ≤ 2 and *j* ≤ 29 would bring to the lower rate value of 6.98 × 10^–17^ cm^3^/s. The tunneling correction factor of ICVT/μOMT in ref (26) is 1.27, and this means that the expected discrepancy between classical and quantum treatment is more than three times higher than the discrepancy observed between present QCT and Sayós et al. data (see also the following discussion for Figures 2 and 3). In Table 1, the QCT result by Caridade and Varandas on their PES^32^ is much lower, but it is obtained as an *extrapolation* from values of temperature of at least 1000 K, so it can hardly be used for comparing the PES and data quality.

**Table 1:** Comparison of the Present Low-Temperature Reactive Result with Data from the Literature (in Parentheses, the Exponents of 10)

rate coeff (cm^3^/s)	*T* (K)	references	method
1.08 ± 0.1(−16)	302	Clark and Wayne 1970^31^	experimental
7.5 ± 0.5(−17)	300	Westernberg et al. 1970^36^	experimental
7.2 ± 0.97(−17)	298	Winkler et al. 1986^29^	experimental
8.8 ± 0.4(−17)	298	Barnett et al. 1987^30^	experimental
7.10 ± 0.26(−17)	300	this work	QCT
7.64(−17)	300	Sayós et al. 2002^26^	ICVT/μOMT
1.75(−17)	300	Caridade and Varandas 2004^32^	QCT extrapolation

In Figure 1, the well-known result of Bose and Candler is also shown,^33^ which is lower (almost a factor 2 at 1000 K) than both the Baulch et al. data^28^ and the present result up to about 12,000 K. Even slightly lower is the recent result by ref (34), obtained by the QCT method on a couple of new ab initio PESs calculated in the same work. In this case, there is a fairly good level of agreement with the experiment in the high temperature range, starting from at least 1000 K (black curve). On the contrary, for lower temperatures, it is clear from Figure 1 that the result from ref (34) is not able to accurately reproduce the experimental data (there is a factor 4.5 of discrepancy with the Baulch et al.^28^ fit at 600 K). This is unexpected, due to the level of theory used in the PES construction. This has been one of the reasons to prefer the relatively old PES set by the Sayós’ group in calculating the whole database in this work. The other reason is in the availability for comparison of many independent theoretical results obtained on the Sayós’ PESs with different methods, as will be shown in the following sections. Another QCT result on a different PES not reported in Figure 1 is from Duff et al.,^35^ which is at least a factor of 2 lower than the Baulch result at about 550 K, converging for higher temperatures.

The present thermal rate coefficient can be reproduced with the following Arrhenius fit
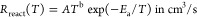
9with the values of the coefficients in Table 2 for two temperature intervals.

**Table 2:** Arrhenius Fit Parameters for the Thermal Rate Coefficient, *R*_react_(*T*), cm^3^/s

temperature range	*A*	*b*	*E*_a_ (K)
300–5000 K	2.726 × 10^–16^	1.505	2989
5000–20,000 K	6.835 × 10^–11^	0.192	9325

In Figure 2, the final vibrational distribution relative to the reactive rate coefficient for N + O_2_ → NO(*v*′) + O at room temperature is shown. It has been obtained as
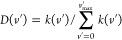
10with *v*_max_^′^(NO) = 47. Final vibrational distribution of the reaction from an initial room temperature equilibrium is particularly important in the chemical kinetics of the thermosphere^6^ because of the vibrationally excited NO emission radiation that can be revealed and can provide detailed data about the thermosphere conditions. This emission is strongly dependent on the vibrational populations of NO, so it is crucial to know this distribution accurately. The best experimental data available are from ref (29), with still unexplained systematically lower values on odd *v* states. The behavior might be due to some quantum effect^29,37^ or due to experimental inaccuracies. However, it is clear in the left panel of Figure 2 that the average experimental trend is well-reproduced by the present quasiclassical calculations. This comparison is particularly good because in both cases, the experimental fluctuations are due to some kind of noise or due to some quantum effect, the classical result should be an average, as it actually is. On the contrary, the comparison with analogous theoretical results obtained with quantum methods^38,39^ appears significantly different from the classical result in the right panel of Figure 2. In fact, both the quantum trends approximate a bell shape centered on *v*′ = 3 or 4, instead of an overall descending trend common to the trends in the left panel. This descending trend is confirmed also by other independent experimental results available^40,41^ (see also Figure S1 in the Supporting Information). The *v*′ = 0 values of the two quantum results are a factor 2 to 4 lower than the experimental data. This can appear surprising because the quantum methods used in refs (38) and (39) are, *in principle*, more accurate than the QCT method of this work. By diving into the details of those calculations, however, it is easy to understand that the specific way in which the methods have been applied are not free from approximations that have a strong impact on the final accuracy of the result.

The two quantum mechanical treatments in refs (39) and (38) differ in the time-dependent/time-independent methods used, respectively, while the J-shifting approximation has been used for computational convenience in both works. It should be also noted that the excited ^4^A′ PES used in ref (39) is slightly different from the one used in ref (38) and in the present work. However, its importance for reactivity at *T* = 300 K is orders of magnitude lower than the result on the ^2^A′ PES. The comparison of the reaction cross sections from *v* = 0, *j* = 1 as obtained in this work by QCT and by ref (39) on the ^2^A′ PES is shown in Figure 3. The two results are very near to each other up to about 1.2 eV of collision energy. From that point on, the quantum result increases in a steeper way with respect to the quasiclassical one. This behavior is confirmed in the comparison between the time-dependent and time-independent quantum method results in ref (38), but actually it is only the confirmation of the trend when J-shifting approximation is used in both cases. More interesting is the comparison with the result from ref (42), shown in the same Figure 3, obtained with a quantum-classical method. Unfortunately, it has been calculated on a different PES with a lower threshold to the reaction, but the qualitative aspect of interest is the cross section tendency to a plateau, an aspect shared with the QCT result but not with the approximate quantum method calculation. Moreover, in this collisional system, the reaction barrier is small (≈0.3 eV) but important at *T* = 300 K, so the presence of tunneling through it should be immediately clear at room temperature as a relevant discrepancy between quantum and classical treatments. This discrepancy is expected to be present in the first values (including zero) of total angular momentum *J* and near the reactive threshold, as well shown in ref (43) for a lighter collisional system. On the contrary, discrepancy is observed at a quite high energy, where QCT reliability increases, while the accuracy of the J-shifting cannot be taken for granted (and where it can have the maximum effect on the partial cross section sum). Independently of all these aspects, in the two refs (38) and (39), the final vibrational distribution is obtained only from a very limited set of initial (*v*,*j*) states. The use of all these approximations can easily explain why the two quantum distributions in Figure 2 are so different between them and also significantly different from the experimental one. Due to the huge (if not unfeasible) computational effort required, the *in principle* accurate quantum methods are then used with so many approximations and limitations that the final result of interest for thermosphere modeling is different from the experimental data to a much larger extent than the quasiclassical result. Generally speaking, collisional systems in which all the atomic species involved have masses heavier than hydrogen do not show significative reaction barrier tunneling for temperatures of the order or higher than 300 K,^44,45^ so the accuracy of QCT for the present reaction cross sections should not be surprising. Some general considerations about the reliability of QCT calculations can be found in ref (22), where different cases (reaction, inelastic, and dissociation processes) are taken into account. Concerning the tunneling correction in Sayós et al. ICVT/μOMT result^26^ in relation to Figure 1, only an *accurate* quantum mechanical calculation can definitely clear this point. Nowadays, it has become feasible to produce at least an accurate *partial* QM result, that is, a J-dependent partial result that can be compared directly with the QCT-equivalent calculation, as in ref (43). This kind of study would be worthwhile both from a theoretical point of view because of the presence of a small reaction barrier but also of heavy atoms and from an applicative perspective, for the large technological interest in air species. From the present study and data available, it appears that tunneling contribution is much less (∼8%) of what expected on the basis of the tunneling correction (27%) of ICVT/μOMT on the same PES.

The result in Figure 2 has been extended to a range of temperature (200–1000 K) typically measured in the thermosphere.^46^ It has been interpolated with a bivariate polynomial as a function of final vibration *v*′ and temperature *T*:  using the coefficients presented in Table S1 in the Supporting Information. The temperature range can be useful in accurate modeling of thermosphere dynamics. In the Supporting Information, a figure is presented (Figure S2) showing the good comparison of data with the present fit and with experimental values.

**Figure 4:**
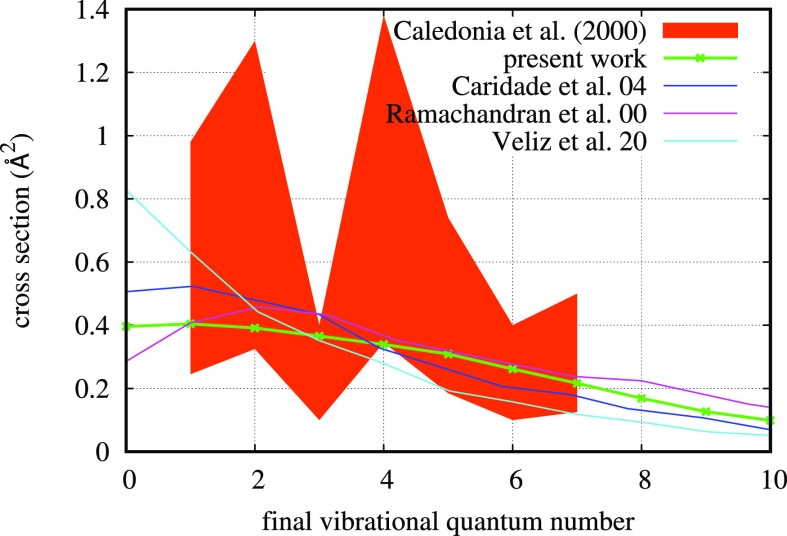
Comparison of the experimental reaction cross section in ref (47) at about 3 eV of collision energy with theoretical result in the present work (green line with markers) and other theoretical data available. The red area indicates the estimated error in experimental values. The rovibrational temperature *T*_vr_ is 300 K; however, the cross section at *T*_vr_ = 1000 K from this work (not shown) is almost coincident.

In Table 3, values and statistical errors of the present results relative to Figure 4 are reported.

**Table 3:** Cross Section and Standard Deviation for the Reaction at 3 eV of Collision Energy as a Function of Final Vibrational Quantum Number, the Same Conditions as in Figure 4

final vibrational quantum number	cross section (Å^2^)	standard deviation (Å^2^)
0	0.395	0.012
1	0.404	0.013
2	0.391	0.012
3	0.365	0.011
4	0.339	0.011
5	0.309	0.011
6	0.261	0.010
7	0.216	0.009
8	0.169	0.008
9	0.127	0.006
10	0.099	0.006

**Figure 5:**
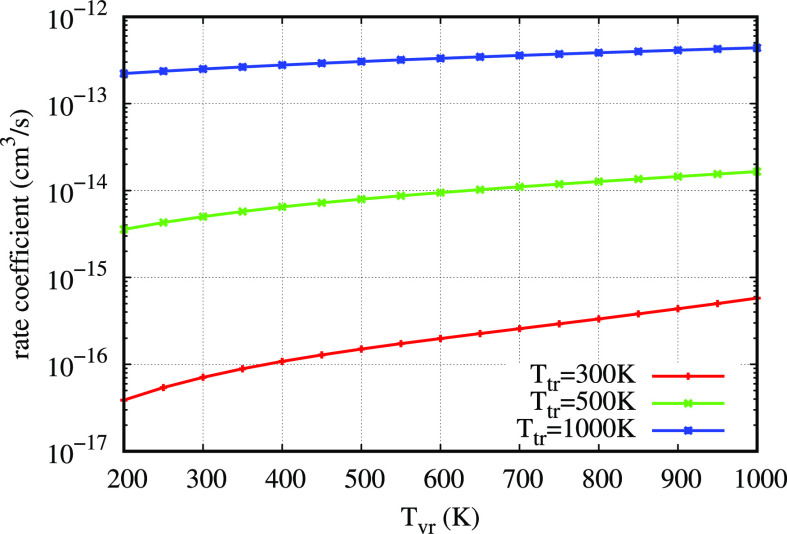
Reactive rate coefficient as a function of rovibrational temperature *T*_vr_ at three values of translational temperature (*T*_tr_) values. The dependence on translational temperature is clearly very high. The dependence on *T*_vr_ is high if *T*_tr_ is low but tends to become less important at sufficiently high *T*_tr_.

In Figure 4, the quasiclassical reactive cross section is compared with the experiment^47^ at high translational energy (3 eV) but low rovibrational temperature (*T*_vr_ = 300 K), as a function of final vibrational quantum number. Even in this case, as for Figure 2, there is a sort of oscillation (with higher values on even vibrational quantum numbers) of the experimental data around the QCT result. Even in this case, the QCT data trend shows a sort of average value of the experimental trend on the whole final vibration range examined. This behavior of QCT results is completely confirmed by similar results from Caridade and Varandas,^32^ Ramachandran et al.,^48^ and San Vicente Veliz et al.,^34^ that we have reported in Figure 4 for comparison (the result of Duff, reported as a private communication in ref (32), is not reported because it is almost coincident with ref (48)). The present calculated cross section at 3 eV of collision energy is almost unchanged when *T*_vr_ = 1000 K, as sometimes used in the literature for this comparison.^32^ This fact, however, does not mean that the reaction process is independent of internal energy. On the contrary, when gas temperature *T*_tr_ is about 300 K (i.e., with translational energy in the range of tens of meV), the rate coefficient dependence on *T*_vr_ is strong, as can be appreciated in Figure 5, with a factor of 15 between the rate coefficient at *T*_vr_ = 300 K and *T*_vr_ = 1000 K. When *T*_tr_ = 1000 K, this factor reduces to 2. Therefore, if translational nonequilibrium is expected to be relevant, using these rate coefficients with only one thermal temperature (i.e., with *T* = *T*_tr_ = *T*_vr_) can result in a very poor approximation in kinetic models.

**Figure 6:**
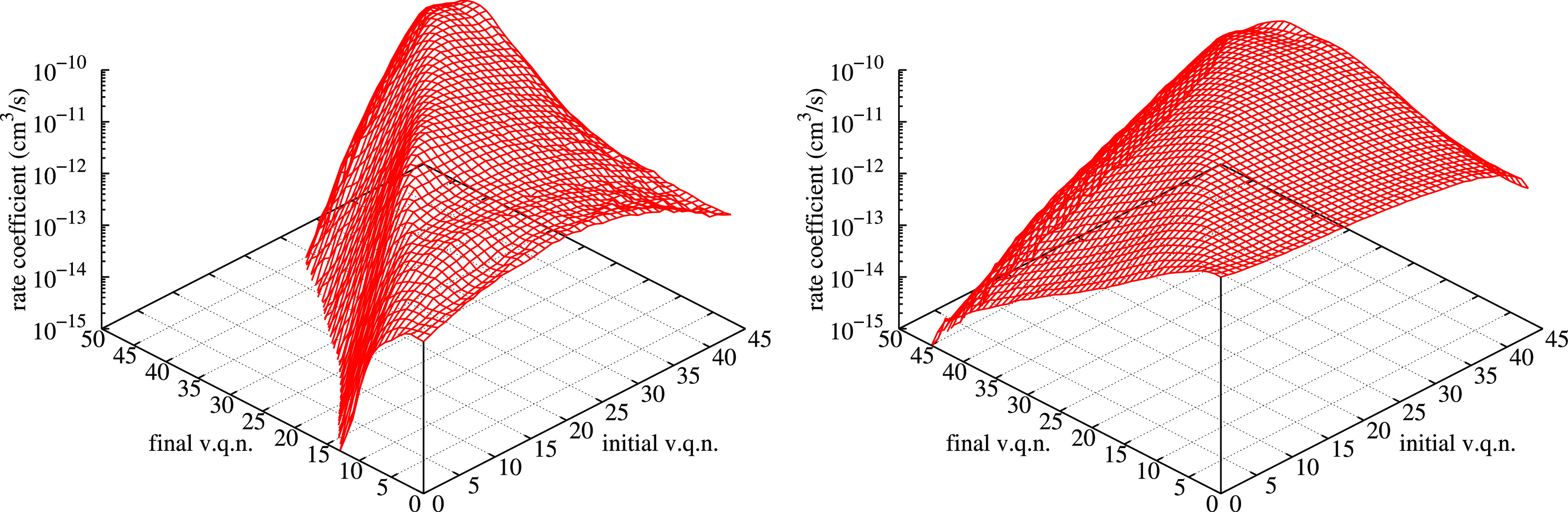
Reactive rate coefficients as a function of initial and final vibrational quantum numbers at two rotranslational temperatures: 2000 K in the left panel and 10,000 K in the right panel.

A global view of the state-to-state reactive rate coefficients as a function of initial *v* and final *v*′ vibrational quantum numbers is presented in Figure 6, *T* = 2000 K (left panel) and *T* = 10,000 K (right panel). The diagonal starting from (*v* = 0, *v*′ = 0) clearly divides the distribution in two sections, the right part of exothermic rate coefficients from the left part of endothermic ones. Exothermic rate coefficients show a variation limited to 1 order of magnitude in the full *v*–*v*′ plane, and even their variation between 2000 and 10,000 K is roughly of the same order of magnitude. On the contrary, endothermic rate coefficients show many orders of magnitude of variation in the same temperature interval. There is a relevant similarity of these reactive rate coefficients with inelastic ones that will be presented and explained in the inelastic processes section below.

### 3.2 Inelastic Processes

The case of inelastic processes should be treated with care, due to an issue intrinsic to the QCT method. This issue is analyzed in refs,^20,23^ distinguishing if the inelastic collision takes place in a purely nonreactive (PNR) or a quasireactive (QR) way. In the first case, the collision is characterized by a weak interaction, with the atom passing by the molecule and only slightly modifying its internal rovibrational motion. In the second case, on the contrary, the interaction is strong and can be thought as a frustrated reaction rather than a simple energy-transfer process. The final vibrational distributions in these two cases are completely different.^21^ In the PNR case, it is characterized by a cusp centered on the value of the initial vibrational state and exponentially decaying, a cusp very narrow at low energy but enlarging as energy increases. It is the typical condition of a Landau–Teller vibrational distribution, or of a forced harmonic oscillator. In the QR case, on the contrary, the initial bond is weakened or temporarily broken, and in the end of the collision, it is rebuilt, but the strong interaction has mixed translational and internal molecular motion. As a consequence, the final vibrational distribution tends to be smooth and flat, with a sort of loss of memory of the initial state, exactly the opposite of the PNR condition. The issue with QCT is limited to low-energy PNR trajectories because standard histogram binning is unable to correctly detect final vibrational actions if the final distribution is too narrow (indeed, *any* kind of binning is unable to do that, including Gaussian binning^49^). On the contrary, QR trajectories, if not affected by significant tunneling through the reaction barrier, are correctly treated by QCT because their vibrational distribution tends to be quite larger than bins in histogram binning. As a consequence, there is a sort of “accuracy threshold” for inelastic processes treated by QCT: below this threshold, the PNR part can be too low or zero, while the QR part tends to follow the same level of accuracy as for the reaction. It essentially shares with reactive events also the same PES region, at variance with low interaction behavior. One can find a striking confirmation in ref (50), where collisions of a different system, O + N_2_, are considered on two ab initio PESs for both reactive and inelastic processes. Reactive rates are in good agreement with results in ref (20), where a different PES set is used, while *total* (PNR + QR) inelastic rates are different by *orders of magnitude* at low energy with respect to the analogous result in ref (20). However, the authors in ref (50) show in their Figure S3 in the Supporting Information that if only the QR contribution is considered, it is in *perfect* agreement with QR in ref (20), confirming that QR and reactive dynamics are quite similar, they share the same PES regions and are deeply different from PNR dynamics.

**Figure 7:**
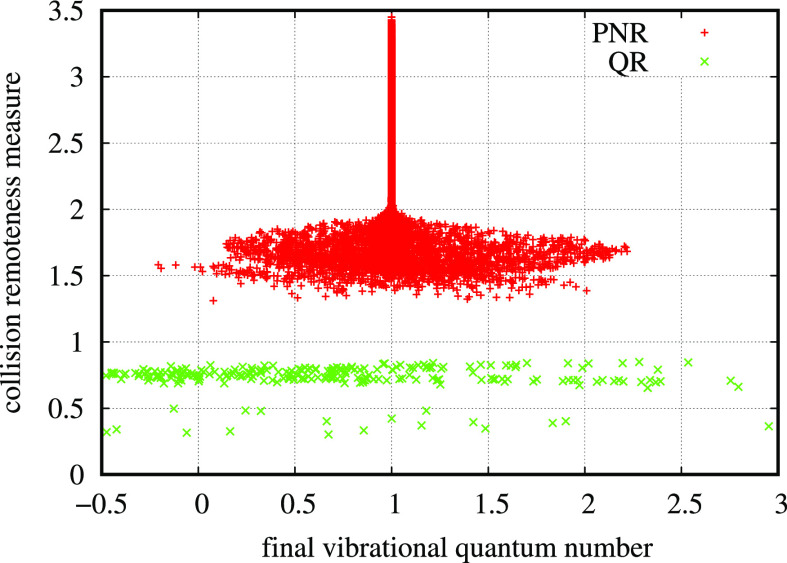
Representation of CRM at collision energy values in the range 0.5–0.6 eV as a function of final vibrational quantum number (indeed a classical continuous number). Each point represents the outcome of a single trajectory. The PNR and QR collisional events generate distributions clearly different and separated in the Figure. The *v*′ = 0 bin ranges classically from −0.5 to 0.5 and clearly includes both PNR and QR trajectories.

The inelastic rate as calculated by QCT is the sum of both PNR and QR parts, but with little effort, it is possible to separate the two contributions, by defining a collision remoteness measure (CRM) attached to each trajectory,^22^ which is an indirect measure of the interaction strength by measuring the lowest “remoteness” of colliding bodies during each whole collision. Considering an atom A colliding with a molecule BC and considering the interatomic distances *R*_AB_, *R*_BC_, and *R*_AC_, the CRM is defined as
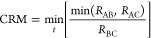
where the first minimum is taken along each trajectory time evolution. CRM is the minimum ratio along a trajectory of the shorter distance of atomic projectile from the BC diatom over the BC distance. For details, the reader is referred to refs.^21,23^ Values of CRM larger than 1 indicate PNR trajectories, while lower values allow to collect QR trajectories (see, in particular, ref (22) for details). The physical relevance of the QCT-poorly approximated PNR contribution depends on the relative weight with respect to the QR part. If this last one is largely predominant (e.g., in the presence of a low reaction barrier), the PNR inaccuracy tends to be negligible. However, it is not obvious to assess this point within the QCT method because PNR contribution can be low for *physical* reasons *or* for the QCT deficiency. For the present collisional system, an analysis has been conducted in this work on the inelastic process from *v* = 1 to *v*′ = 0. Being the lowest exothermic vibrationally inelastic process, it is the most prone to PNR issue at low collision energy because of the low total energy involved. Figure 7 shows the CRM values of a small bunch of trajectories on the ^2^A′ PES relative to a collision energy interval of 0.5–0.6 eV, with an impact parameter range from 0 to 5 Å. CRM is represented as a function of the final vibrational quantum number (indeed a continuous classical quantity). This representation allows us to effectively visualize the two completely different distributions originated from PNR and QR events, respectively. For CRM ≫ 1, the final vibrational action is practically that of the incoming channel, that is, the collision has produced an elastic process. Incidentally, it is useful to remind that a classical elastic process is without convergence, unless a cutoff criterion is introduced. The QM criterion in ref (51) has been partially applied to some subsets of the present calculations, with good results. A specific work will be presented about this topic, of large interest in the direct simulation Monte Carlo applications,^52,53^ due to the need of using consistent sets of cross sections, including the elastic ones. For CRM < 2, also the width of the distribution in Figure 7 starts increasing. Only when its borders are beyond the limits of the initial vibrational bin (i.e., less than *v* – 0.5 or larger than *v* + 0.5, with *v* = initial vibrational state), then QCT can detect a nonzero inelastic probability, which becomes more and more reliable for higher energy values. However, a “barely in the box” probability is generally a poor approximation indeed, as shown in ref (22). With CRM, one can easily separate the PNR from QR distributions and then study their features. In Figure 7, it is easy to distinguish, above CRM = 1 value, the bell-shaped distribution of PNR events from the flat and wide distribution of QR ones, placed well under CRM = 1. For the case shown, it is plain that QR will be correctly detected by QCT binning, while the PNR distribution width is just the minimum for an accurate result, as obtained in ref (22) for another (much lighter) system. This minimum has been empirically determined as the condition in which the distribution at least “touches” the center of the final bin. In ref (22), there is an accurate quantum mechanical result to compare with. However, more investigations are required to assess this important point with different systems and conditions. For higher total energy, the reliability rapidly tends to improve, and as a consequence, it is very likely that for collision energy values larger than about 0.5 eV, all the other inelastic results are accurate (or at least this *necessary* condition for accuracy is surely satisfied). On the other hand, QR contribution in the same conditions is quite high (as can be inferred from the QR point density near *v*′ = 0) due to the low barrier, so probably even some inaccuracy in PNR should not be of great relevance with respect to the QR probability magnitude.

Concerning the ^4^A′ PES, where the reaction barrier is higher than the one of ^2^A′ by about 0.3 eV, qualitatively similar results have been obtained but at slightly higher energy values. As a consequence, a temperature of 1000 K is probably a good lower limit for QCT-treated inelastic processes for the present collisional system. It is worthwhile to stress that this accuracy lower limit is much lower than the one in the O + N_2_(*v* = 1 → *v*′ = 0) inelastic process with similar masses. In that case, the system is not reactive for more than 3 eV of collision energy, so all the worst QCT features are present in the inelastic rate.^20^ In fact, the O + N_2_ QCT inelastic cross section for the cited transition is completely zero under 1.8 eV, at variance with more accurate calculations.

**Figure 8:**
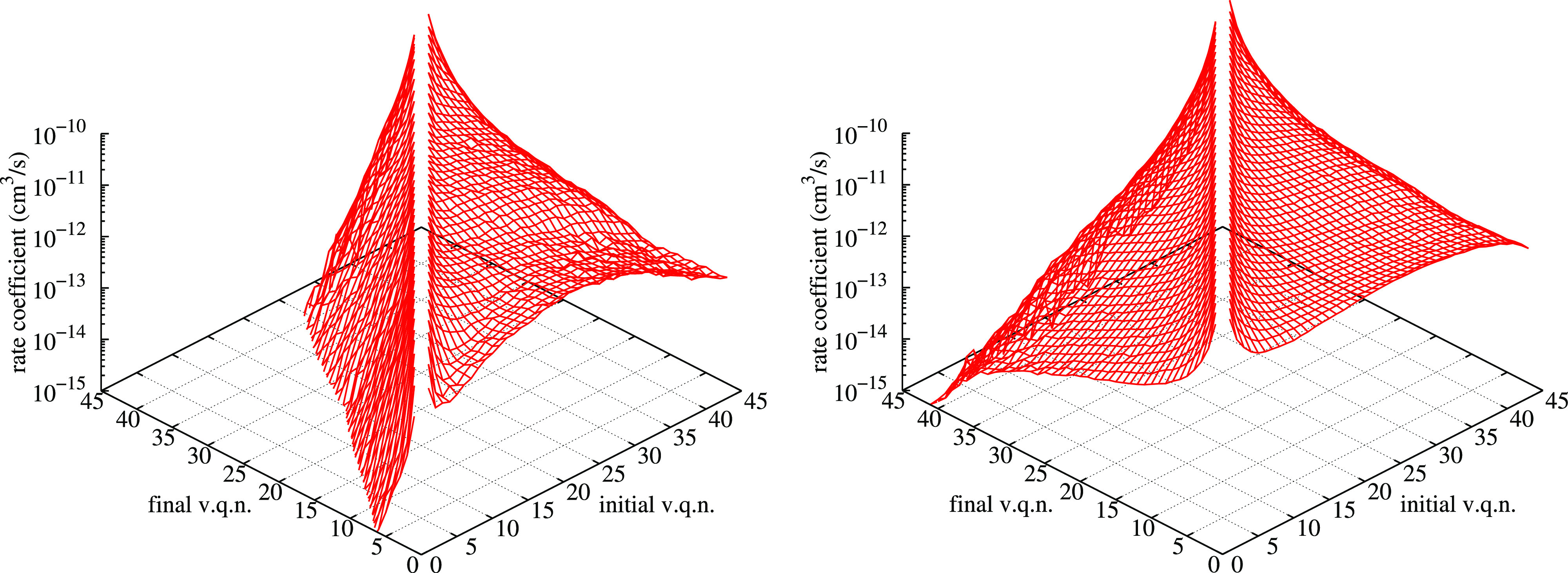
Inelastic rate coefficients as a function of the initial and final vibrational quantum numbers at two temperatures, *T* = 2000 K in the left panel and *T* = 10,000 K in the right one.

The rate coefficients obtained by integrating the corresponding cross sections calculated in this work on both PESs with the usual degeneracy factors are shown in Figure 8 at two temperatures, 2000 K in the left panel and 10,000 K in the right one, as a function of both initial and final vibrational quantum numbers. The right “wing” of each surface is of exothermic rate coefficients, while the left wing is made of endothermic rate coefficients. The surfaces are quite smooth, due to the high number of trajectories used in this work. The diagonal of elastic processes is omitted for the reasons already presented.

In this figure, it is interesting to appreciate the different behaviors of PNR and QR processes. PNR typical behavior can be seen near the diagonal of each picture, where the rate coefficients show a systematic cuspidal trend around the (missing) elastic rate coefficient. On the contrary, the rest of the rate coefficients, made essentially of QR processes, is generally smooth and constitutes the bulk of the whole distribution. It is interesting to also stress the trend similarity of this figure, once the quasidiagonal peaks are neglected, with the one in Figure 6, due to the common features of reactive and QR processes. It is important here to stress that QR rate coefficients in the right wing are not negligible at all in comparison with PNR ones, in particular if nonequilibrium conditions must be studied in the kinetics. In the past, many approximate dynamical methods based on a forced harmonic oscillator model have allowed the compilation of databases in which only the PNR contribution can be approximately taken into account, neglecting the QR one. Now that QCT calculations are affordable on large supercomputers, the major contribution of this method is to provide the whole picture of the rate coefficients including QR transitions, provided PNR contribution is correctly treated. Another interesting possibility, currently actively investigated in this group,^22^ is to extract only the QR contribution from QCT at low energy and then adding it to the PNR contribution from other suited semiclassical models, in which QR contribution could be unreliable. Some preliminary results are very encouraging and will be published soon. Even in this case, the comparison with accurate QM calculations would be important for assessing the accuracy of the results, while experimental data are missing.

### 3.3 Dissociation

#### 3.3.1 Spin–Orbit PES Degeneracies

When calculating dissociation rate coefficients of N + O_2_ from electronic ground-state reagents, one should consider the sum of dissociation from all the relevant PESs involved in the process, with the suited degeneracy factors due to the multiplicities associated with each PES, that is, in the specific case, all spin–orbit combinations of N(^4^S) and O_2_(^3^Σ_g_). This means considering ^2^A′, ^4^A′, and ^6^A′ PESs, with degeneracy factors obtained as ratios of each PES degeneracy to total degeneracy: 2/12, 4/12, and 6/12 for the three PESs, respectively. There is no significant temperature dependence of the spin–orbit degeneracies to consider for the range of temperatures of the present study (see also Sayós et al.^26^ and ref (54)). This is true both for internal energy exchange processes as well as for dissociation. However, for dissociation, there is an important approximation that can be exploited and that only recently can rely on comparisons with complete calculations concerning O + O_2_. The simplification consists in considering dissociation as approximately independent of the specific PES, that is, approximately equal on each PES in the set. This means that the spin–orbit dissociation summed rate coefficient
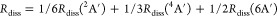
11with
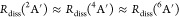
12is simply given by dissociation on, for example, the ground PES, *R*_diss_(^2^A′), without any degeneracy factors. Actually, dissociation rate coefficients show trends quite similar across different PESs of the same system (indeed, even across different collisional systems, although to lesser extent, see the general discussion in ref (55) and the application in ref (56)). The deep reason is that the process takes place at an energy normally quite higher than the typical level at which all the subtle differences among different PESs are of relevance. On the contrary, PES features that matter for the dissociation process are not so different from one PES to the other, so in a first approximation, they can be ignored and dissociation can be obtained on the most accurate PES available, considering all the other PES contributions by simply avoiding any degeneracy factor. This approximation has been implicitly used, for example, in ref (57) (apart from the Nikitin factor, discussed in the next section). Two recent, completely independent works about O + O_2_ dissociation can now quantitatively support these observations. Both in refs (58) and (59), all the nine PES spin–orbit correlated to the same ground-state reagents in O + O_2_ collisions have been independently calculated with accurate ab initio methods and then dissociation has been obtained with the quasiclassical method using all the PESs, with the suited degeneracy weights.

In ref (58), a direct comparison of thermal dissociation is shown with the results from ref (57), where only the ground-state PES has been used and no degeneracy factor has been applied. The comparison is very good in the whole temperature range explored. In the second case,^59^ dissociation from each PES is available, and it is clear that differences among different PESs have a very limited relevance for dissociation (unfortunately, this is not the case for internal energy-transfer processes, that appear to be strongly influenced by specific PES features, as expected and also shown in ref (59)). As a consequence, it is more than plausible that also in the N + O_2_ case, dissociation behaves in a similar way across the multiple PESs in principle available for dissociation (but much less studied than for the O + O_2_ case). To support this observation, in Figure S3, the comparison of dissociation rate coefficients *R*_diss_ calculated on ^2^A′ and ^4^A′ in this work is presented. In order to stress the uniform ratio of the curves as a function of the initial vibrational quantum number, the *R*_diss_ on the ground-state PES has been multiplied by 0.75. Considering that the ^6^A′ PES is not available, the possibility of approximating it by the results obtained on the other PESs is crucial. In this work, we have chosen to put the dissociation on ^6^A′ PES equal to the average value obtained on the two lowest PESs
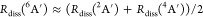
13

Taking into account the previous observations, the mean value (*R*_diss_(^2^A′) + *R*_diss_(^4^A′))/2 is now considered as the approximate value of dissociation, irrespective of degeneracy factors, concerning all the spin–orbit-correlated PESs of N + O_2_. In the next section, the problem of approximating dissociation also from excited electronic states of O_2_ will be treated.

#### 3.3.2 Nikitin Factor

In the text of Nikitin,^60^ there is a proposal of a model that takes into account the presence of excited electronic states of the O_2_ molecule in the collision-induced dissociation with another atom or molecule. The problem is particularly important because the energy involved can be of the order of several eV (about 5.1 eV from *v* = 0, *j* = 1), so it is quite likely that the process is significantly affected by the presence of reagent excited states that can even be as low as 1 eV from the ground electronic-state curve (ground e-state). The idea is that there is an equilibrium between the vibrational states (*v*-states) of the ground e-state and the corresponding *v*-states with a similar energy relative to excited e-states. Once this hypothesis is verified (see below), one can consider the contribution to dissociation from each excited e-state as the ground e-state dissociation times the ratio of the degeneracy of the excited e-state with respect to the ground e-state degeneracy (a triplet). As a consequence, dissociation including O_2_-excited contributions will be calculated as the ground e-state dissociation rate coefficient times the degeneracy sum over all the e-states considered divided by the ground e-state degeneracy (see ref (57) for details about this point). This model is presented by Nikitin with reference to two different groups of O_2_ states, the lowest e-states (a^1^Δ_g_ and b^1^Σ_g_^+^) and the Herzberg states (c^1^Σ_u_^–^, C^3^Δ_u_, A^3^Σ_u_^+^). Following Nikitin, for the low-lying states, the equilibrium is probably slow. We can add qualitatively that this is the case because the Franck–Condon factors for the radiation-less transitions are generally quite low for each couple of *v*-states in different e-states and similar total energy, also considering the almost coincidence of the e-states minima (see Figure S4 with its caption, and the discussion in ref (61), p 327). However, also dissociation from those low e-states is quite low, so it is possible that an equilibrium is established. If *v*-states near dissociation are considered, on the contrary, there are many possible intersections of e-states (also including the lowest ones), continuously modified as the third body approaches the diatom. Consequently, a vibrational oscillation in one of the e-states has a large probability of nonadiabatic transition toward any other e-state, including also lower e-states. This is plausible because all the e-states converge in the classical maximum vibrational elongation along the ground e-state, as clear from Figure S4. A support to these observations comes from the studies about PESs in O_3_^62^ and NO_2_^63^ photodissociation. It is very likely that the nonadiabatic rate of transition can be significantly higher than the dissociation from the ground e-state; consequently, a further channel of parallel dissociation is provided, with the suited degeneracy of the electronic state involved. From the discussion in the previous section, it should be clear that dissociation is not very sensitive to PES features. Consequently, analogously to approximating dissociation from spin–orbit-correlated PESs with dissociation from only ground-state PES, one can consider dissociation from diatomic excited e-states (with the appropriate PESs, in part still approximately or not known, especially for N + O_2_) as quantitatively similar to that from the ground e-state, with the appropriate degeneracy.

**Figure 9:**
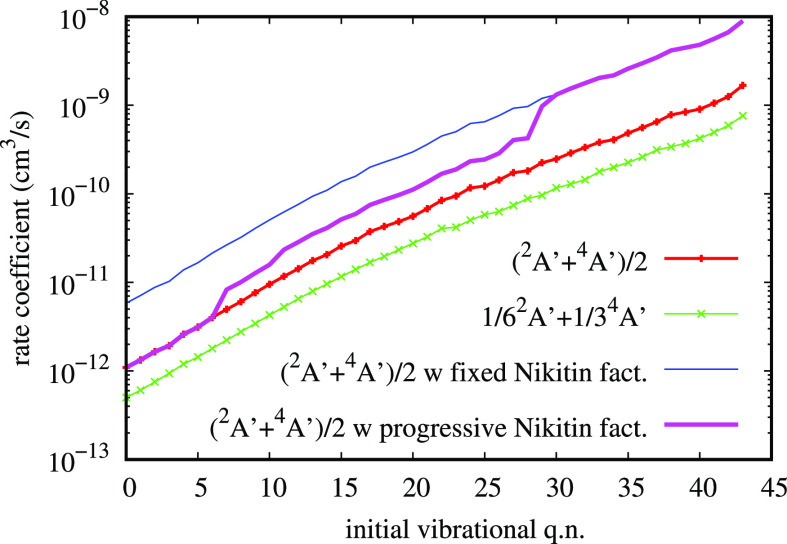
Comparison of dissociation rate coefficients at *T* = 10,000 K as a function of the initial vibrational quantum number, as obtained considering the standard sum with degeneracy factors, the average value on the first two PESs, the same with the fixed and progressive Nikitin factors (see text). It is worth noticing the increasing “steps” along the horizontal axis in the progressive Nikitin curve, due to the approximate inclusion of contributions from more O_2_ electronic states, as the initial *v*-state increases.

**Figure 10:**
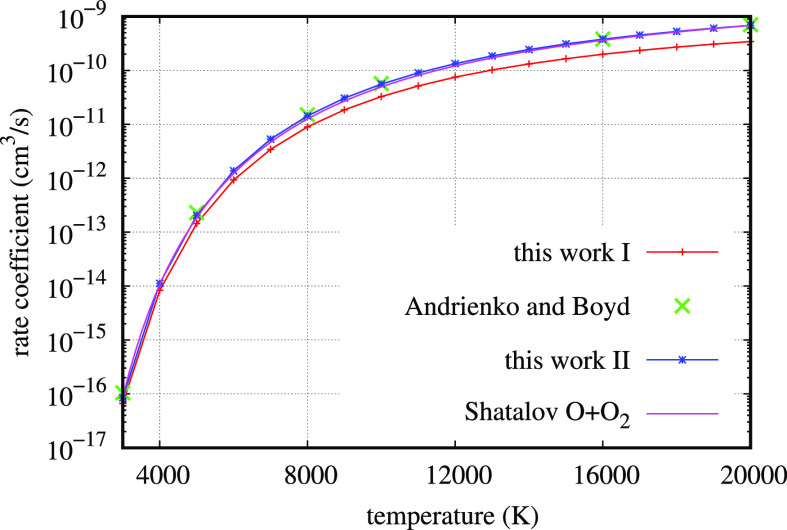
Comparison of the thermal dissociation rate coefficient calculated as explained in the text (this work I), as calculated in ref (67) (Andrienko and Boyd) and similarly recalculated in this work (II), with experimental result in ref (68) (Shatalov O + O_2_). All the results agree on the whole temperature range (see text for comments).

Considering that obviously, it is impossible to find *v*-states with similar energy belonging to ground and excited e-states if the *v*-state relative to the ground e-state has an energy lower than the minimum of the excited e-state, a suitable modification of the original Nikitin factor has been formulated in a paper of one of us in 2002,^57^ consisting in applying the Nikitin factor by considering the multiplicities only of e-states having a minimum lower than the considered reagent *v*-state of the ground e-state. This produces a sort of *progressive* Nikitin factor that increases with the energy of the initial *v*-state. This progressive model is particularly relevant when applied in contexts in which state-selected rate coefficients are required for kinetic simulations. On the contrary, the original Nikitin model was applied to a thermal rate coefficient, so in that context, it is perfectly justified, but it is *not* alternative to the progressive version, in the sense that for state-selected rate coefficients, only the progressive model makes sense. The difference in the final value of thermal rate coefficient using the fixed (original) Nikitin factor or the progressive one is relatively small (also see the discussion on Figure 10 below), due to the relative low relevance of dissociation in the Boltzmann sum from low-lying *v*-states. In general, however, it is not so for state-to-state models. The impact in using the progressive factor is shown in Figure 9, where the dissociation rate coefficient at *T* = 10,000 K is presented as a function of the initial vibrational quantum number. The lowest curve is obtained by summing the contributions from ^2^A′ and ^4^A′, weighted with the appropriate degeneracy factors. In this way, there is no contribution from ^6^A′. The red curve is obtained by a simple average between ^2^A′ and ^4^A′ results, on the basis of the previously presented idea of considering approximately equal the contributions from the *three* PESs. The blue curve is obtained from the same simple average of ^2^A′ and ^4^A′ results with a fixed Nikitin factor of 16/3. The pink curve is based on the same average but using the progressive Nikitin factor, as clear from the presence of some “steps” in the curve. For clarity, a table of progressive Nikitin factor is provided in Table S2 of the Supporting Information. There is also a difference with original application of the “variable” Nikitin factor in ref (57), where the factor is the same but applied considering the energy of rovibrational states, instead of the vibrational state, as in this work. The present version is based on the consideration that rotational potential in the diatom approximately changes all the vibrational levels of the same amount, so all the e-state minima are increased more or less of the same amount as the vibrational levels in the ground e-state. This means that the progressive factor procedure is not significantly changed by rotation.

It is also important to stress that if the Nikitin factor works well for O + O_2_, as clear from different, independent works (see refs (57)−^59^, (64), and (65)), it should be correct also *for any other projectile* against O_2_, such as Ar, O_2_, and N. In fact, all the Nikitin’s observations are related to electronic states of O_2_, largely irrespective of the nature of the third body in the collision. For O_2_–O_2_, there is a direct evidence from the recent calculations of Grover et al.^66^ For Ar–O_2_, the same Nikitin^60^ has successfully applied his original model to that case; therefore, it is very likely that the behavior of N + O_2_ dissociation will be quite like that of O + O_2_.

#### 3.3.3 Accuracy of the Dissociation Result

Unfortunately, there is no experimental data for a direct comparison with the calculated dissociation rate coefficients. What is sometimes found in the literature is the O + O_2_ dissociation rate coefficient,^68,69^ that in the absence of any other evidence is used also for N + O_2_. The two collisional systems are expected to share some features, so it stands to reason to expect similar values of dissociation. Even considerations from the classical impulsive model for dissociation^53^ qualitatively confirm this expectation. Figure 10 represents the thermal equilibrium dissociation rate coefficient for N + O_2_ collisions as obtained in this work with a progressive Nikitin factor (“this work I”), compared with the experimental data from ref (68) relative to O + O_2_. The other curves in the figure are the N + O_2_ dissociation result from Andrienko and Boyd,^67^ and the dissociation obtained by the present calculations in order to mimic their result (“this work II”). They propose to consider the ^6^A′ contribution as two times the dissociation from ^2^A′ and ^4^A′ summed with the appropriate degeneracy factors 1/6 and 1/3, respectively, and then, they apply^70^ the fixed Nikitin factor (16/3). While the agreement of “this work II” with Andrienko and Boyd is excellent, as well as the agreement with O + O_2_ experimental dissociation rate, the present result “this work I” with the progressive Nikitin factor is slightly lower, as expected from the application of the model and the results in Figure 9. Generally speaking, all the results are clearly in good agreement, reinforcing the feel that collisional dissociation seems to show a weak dependence not only on the specific PES used but also on the specific collisional system. However, O + N_2_ dissociation shows only a qualitative agreement with N + N_2_ experimental dissociation in ref (20), with a typical discrepancy within about 1 order of magnitude. Therefore, the present comparison with the O + O_2_ experiment remains qualitative in nature. The thermal dissociation rate coefficient from this work (I in Figure 10) can be easily reproduced with the following Arrhenius equation
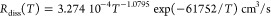
14

The whole set of vibrational state-selected dissociation rate coefficients as a function of the initial vibrational quantum number and roto-translational temperature is available by means of the data file in the Supporting Information.

From general considerations on the QCT method,^22^ it is possible to estimate the level of reliability of the dynamical method used for these calculations. As well-known,^71^ a practical indicator for QCT dissociation reliability is the discrepancy between the dynamical threshold (the threshold obtained from the dynamics) and the theoretical energy threshold expected for dissociation. In the case of He + H_2_ collisions, this discrepancy is of the order of 0.7 eV or worse.^22^ On the contrary, for H + HeH^+^ collisions, this discrepancy is of the order of only 0.05 eV.^24^ In fact, the reason for the QCT dissociation failure is not necessarily linked to the low masses involved, but it is due to more complex mechanisms.^72^ For the present system, this discrepancy is of the same order of the energy axis discretization (0.1 eV in this work for temperatures higher than 1000 K), that is, it is the best possible. Here, one can repeat the same considerations made in ref (20), where the presence of open reactive and inelastic channels at the dissociation energy threshold is an important prerequisite for reliable QCT results. More specifically, in ref (22), a subtle distinction between “inelastic” and “reactive” dissociation is made, showing that even in this case, the QCT response to the two processes can be quite different. In the first case, the collisional system is dissociating by compression of the initial molecular bond in an inelastic way, in the sense that in the reagent molecule, vibration becomes so energetic that breaks the diatom. Dissociation can be seen in this case as a further vibrational level beyond the maximum, and the whole process is a purely inelastic one, subject to the same QCT limitations already seen for PNR contributions. Inelastic contribution to dissociation is expected to be generally low, but in nonreactive collisional systems, it can be the only contribution, so QCT inaccuracy in the threshold region becomes important in a relative sense. However, if the system is reactive, even the reactive contribution to dissociation comes into play, and it is generally largely predominant over the inelastic one because in this case, there is also an attractive mechanism for dissociation, accompanied by a strong interaction. This explains the good QCT dissociation performance on reactive systems and the quite limited success for inelastic systems at total low energy (see ref (22) for details). Another source of dissociation is from collisions with rotational quasibound states,^72^ but for heavy species, its contribution is quite limited, as already discussed in ref (20), due to the long lifetimes of quasibound states. Summarizing, the QCT method is expected to be accurate in the determination of dissociation for the present collisional system. Of course, the final result is necessarily the composition of many different aspects, such as the PESs used, the hypothesis on the third PES ^6^A′, the treatment of the O_2_ excited electronic states, and the dynamical method used.

## 4 Conclusions

In this work, the collisions of atomic nitrogen with molecular oxygen have been studied, including reactive, inelastic, and dissociation processes, considering the whole vibrational ladders of reactants and products. Rate coefficients for all these processes have been obtained by integration of the corresponding cross sections, calculated in this work by QCT in the collision energy range 10^–3^–10 eV. The capabilities and limits of the adopted dynamical method have been discussed for each kind of process, giving comparisons with experimental and other theoretical data, when possible. From the present analysis, it appears that the results obtained are at least very reliable (very accurate in the case of reaction) on the whole temperature range for which a comparison is available. Suggestions for some specific QM studies are formulated, in particular, in order to assess the relevance of the tunneling for the reaction, which appears to be of limited relevance, as well as the lower limit of applicability of QCT in the vibrationally inelastic processes for the present collisional system at room temperature.

A particular interpolation fit tailored on the needs in thermosphere kinetics is obtained, based on the present calculations. Detailed rate coefficients calculated in this work are available in the Supporting Information. A forthcoming paper will be dedicated to the interpolation of all the detailed rate coefficients presented here, in order to make them fully available in the chemical kinetics community.

## Supporting Information Available

Coefficients of polynomial fit of the decimal logarithm of reactive rate coefficients in the temperature range 200−1000K; comparison of reaction rate coefficients with other experimental data; comparison of reactive rate coefficients as a function of final vibrational quantum number; comparison of dissociation rate coefficients as a function of initial vibrational quantum number; electronic states of O_2_ of interest in the text; and progressive Nikitin factor in the dissociative reaction (PDF)
Data file with reactive vibrational state-to-state rate coefficients at different values of roto-translational temperature (TXT)
Data file with inelastic vibrational state-to-state rate coefficients at different values of roto-translational temperature (TXT)
Data file with dissociation vibrational state-to-state rate coefficients at different values of roto-translational temperature (TXT)

## Dedication

The authors dedicate this work to the memory of their respective parents.
