# Supplementary material for: Reactive, Inelastic, and Dissociation Processes in Collisions of Atomic Nitrogen with Molecular Oxygen
Source: J Phys Chem A. 2021 Apr 28;125(18):3953–64. doi: 10.1021/acs.jpca.0c09999 (PMC9282678; doi:10.1021/acs.jpca.0c09999)
Supplement: Supplementary file 1 — jp0c09999_si_001.pdf [file jp0c09999_si_001.pdf]

# Reactive, Inelastic, and Dissociation Processes in Collisions of Atomic Nitrogen with Molecular Oxygen

Fabrizio Esposito*[,1], Iole Armenise*[,1]

[1] *CNR ISTP, Via Amendola 122/D, 70126 Bari, Italy*

E-mail: fabrizio.esposito@cnr.it, iole.armenise@cnr.it

## Supporting Information

**Table S1.** Coefficients of polynomial fit of the decimal logarithm of reactive rate coefficient (in $cm^3/s$) as a function of final vibration in the range v'=0-9 and temperature in the range 200K-1000K:

$f(v',T) = \sum_{i,j=0-5} a_{ij} v'^{i} T^{j}$, $R_{react}(v',T) = 10^{f(v',T)}$

with:
a00 = -31.7856
a01 = -2.38785
a02 = 2.51941
a03 = -0.915124
a10 = 0.103946
a11 = 0.014007
a12 = -0.0146947
a13 = 0.0052902
a20 = -0.000273671
a21 = -3.93603e-05
a22 = 4.08553e-05
a23 = -1.46132e-05
a30 = 3.851e-07
a31 = 5.76655e-08
a32 = -5.97883e-08
a33 = 2.129e-08
a40 = -2.74532e-10
a41 = -4.19722e-11
a42 = 4.3754e-11
a43 = -1.55547e-11
a44 = 2.28945e-12
a04 = 0.137229
a14 = -0.000790635
a24 = 2.1713e-06
a34 = -3.14553e-09
a50 = 7.78476e-14
a51 = 1.19948e-14
a52 = -1.26009e-14
a53 = 4.48031e-15

a54 = -6.58062e-16
a55 = 3.45072e-17
a05 = -0.0073388
a15 = 4.21429e-05
a25 = -1.15166e-07
a35 = 1.65981e-10
a45 = -1.20345e-13

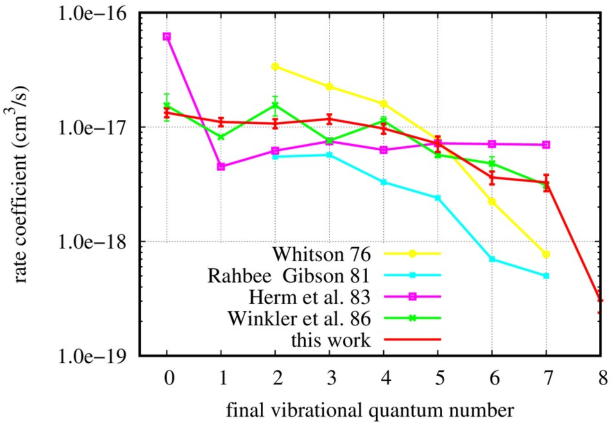


***Figure S1.*** *Comparison of reaction rate coefficients of N+$O_2$→NO(v')+O at T=300K as obtained using the QCT method (this work, with error bars) and the experimental results by Whitson[1], Rahbee and Gibson [2], Herm et al.[3] and Winkler et al.[4](with error bars).*

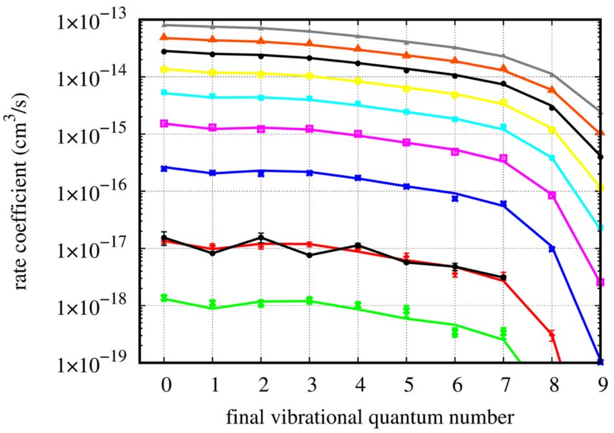


***Figure S2.*** *Comparison of reactive rate coefficients (points) as a function of final vibrational quantum number at different temperatures with the interpolation provided in this work (curves). The points and related curves are relative to T=250K (the lowest, green one), then at steps of 100K from 300K up to 1000K. At 300K even the experimental values from ref.*[4] *is shown (black thick curve).*

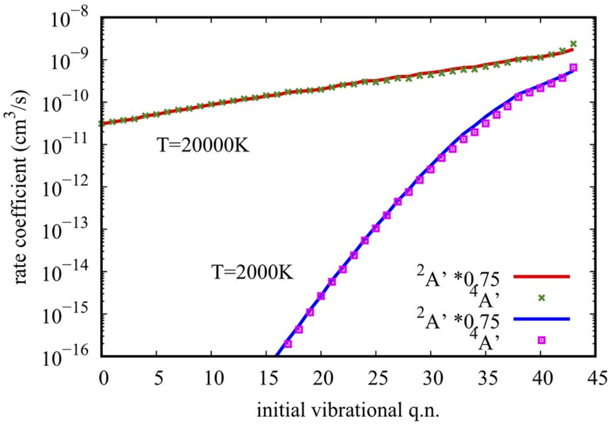


***Figure S3****. Comparison of dissociation rate coefficients as a function of initial vibrational quantum number calculated in this work on the $^2$A' and $^4$A' PESs, at two temperatures. On the $^2$A' PES the result has been multiplied by 0.75.*

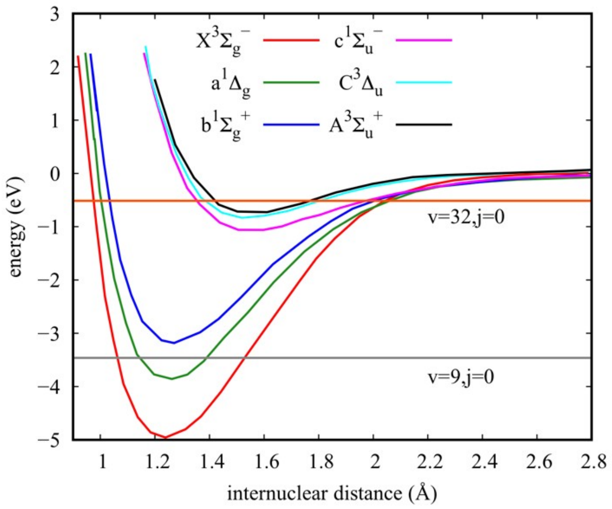


***Figure S4****. Electronic states of $O_2$ of interest in the text. For the lower v-states of the ground e-state the equilibrium can possibly be established only with low-lying e-states showing at least a minimum not higher than the v-state energy. The consideration of this necessary condition does not change the thermal rate significantly, but is of importance when dealing with state-selected dissociation rate coefficients. The curves are taken by hand from[2]. The ground e-state in that reference appears too high by about 0.3eV with respect to the value found in this work, but it is not relevant in the model application. In the examples in the figure, v=9 of the ground e-state has an associated Nikitin factor of (3+2)/3, being 2 the degeneracy of $a^1\Delta_g$. For v=32, the maximum value is obtained: (3+2+1+1+6+3)/3 = 16/3.*

**Table S2.** Progressive Nikitin factor in the dissociative reaction N + $O_2$(v) --> N + O + O

| vibrational levels (of the ground electronic state) | Nikitin factor |
|---|---|
| v ≤ 6 | 1 |
| 7 ≤ v ≤ 10 | $\frac{5}{3}$ |
| 11 ≤ v ≤ 26 | 2 |
| 27 ≤ v ≤ 28 | $\frac{7}{3}$ |
| 29 ≤ v ≤ 29 | $\frac{13}{3}$ |
| 30 ≤ v | $\frac{16}{3}$ |

**References**


(1) Whitson, M. E.; Darnton, L. A.; McNeal, R. J. Vibrational Energy Distribution in the NO Produced by the Reaction of N(4S) with O2. *Chemical Physics Letters* **1976**, *41* (3), 552–556. http://dx.doi.org/10.1016/0009-2614(76)85415-2.
(2) Rahbee, A.; Gibson, J. J. Rate Constants for Formation of NO in Vibrational Levels v = 2 through 7 from the Reaction N(4S)+O2→NO‡+O. *The Journal of Chemical Physics* **1981**, *74* (9), 5143. https://doi.org/10.1063/1.441723.
(3) Herm, R. R.; Sullivan, B. J.; Whitson, M. E. Nitric Oxide Vibrational Excitation from the N(4S)+O2 Reaction. *The Journal of Chemical Physics* **1983**, *79* (5), 2221. https://doi.org/10.1063/1.446071.
(4) Winkler, I.; Stachnik, R. A.; Steinfeld, J. I.; Miller, S. M. Determination of NO (V=0–7) Product Distribution from the N(4S)+O2 Reaction Using Two-Photon Ionization. *The Journal of Chemical Physics* **1986**, *85* (2), 890. https://doi.org/10.1063/1.451840.
(5) Saxon, R. P.; Liu, B. Ab Initio Configuration Interaction Study of the Valence States of O2. *The Journal of Chemical Physics* **1977**, *67* (12), 5432–5441. https://doi.org/10.1063/1.434764.
